# Characteristics of Sonography in a Rat Achilles Tendinopathy Model: Possible Non-invasive Predictors of Biomechanics

**DOI:** 10.1038/s41598-017-05466-y

**Published:** 2017-07-11

**Authors:** Su-Ya Lee, Hsiao-Feng Chieh, Chien-Ju Lin, I-Ming Jou, Yung-Nien Sun, Li-Chieh Kuo, Po-Ting Wu, Fong-Chin Su

**Affiliations:** 1 Department of Biomedical Engineering, National Cheng Kung University, Tainan, Taiwan; 2 Medical Device Innovation Center, National Cheng Kung University, Tainan, Taiwan; 3 Department of Orthopedics, E-Da Hospital, Kaohsiung, Taiwan; 4 Department of Orthopedics, College of Medicine, National Cheng Kung University, Tainan, Taiwan; 5 Department of Computer Science & Information Engineering, National Cheng Kung University, Tainan, Taiwan; 6 Department of Occupational Therapy, National Cheng Kung University, Tainan, Taiwan; 7 Department of Orthopedics, National Cheng Kung University Hospital, College of Medicine, National Cheng Kung University, Tainan, Taiwan

## Abstract

The purpose of this study was to investigate the dynamic changes of histopathology, biomechanical properties, echo intensity, and ultrasound features in a collagenase-induced tendinopathy model of rat Achilles tendons, and to examine the associations among biomechanical properties, echo intensity, and ultrasound features. Forty-two rats received an ultrasound-guided collagenase injection on their left Achilles tendons, and needle puncture on the right ones as the control. At four, eight, and twelve weeks post-injury, the tendons were examined via measurements of their biomechanical properties, histopathological and ultrasonographic characteristics. The injured tendons showed significantly higher histopathological scores, lower Young’s modulus, and higher ultrasound feature scores than the those of control ones throughout the study period. Up to *week 12*, all injured tendons showed defective healing. The neovascularization score had a significant negative linear association with the failure stress and Young’s modulus. Maximum normalized echo intensity had a significant positive linear association with maximum strain. Therefore, neovascularization and maximum normalized echo intensity are associated with mechanically altered tendinopathic tendons. Non-invasive ultrasound methodology, including echo intensity and ultrasound feature scores, may provide useful information about biomechanical properties of tendinopathic tendons.

## Introduction

Tendinopathy is the most common soft tissue injury^1^, but its pathogenesis remains unclear. In order to thoroughly investigate the pathogenesis and management strategies of tendinopathy, the use of animal models^2–5^ is crucial as they provide details of various evaluative aspects of tendinopathy, such as morphology, histology, and biomechanics. Collagenase-induced tendinopathy, also called calcified tendinopathy, is a commonly used experimental model with well-established histopathological evidence^6, 7^. However, few studies have examined the changes in biomechanical behavior during healing in order to understand the pathology and functional prognosis of collagenase-induced tendinopathy in more detail. Understanding the dynamic changes in biomechanical properties is essential to investigating the process of tendon healing, and to provide better approaches for dealing with tendinopathy.

Clinically, the ultrasound (US) assessment of tendinopathy is increasingly being employed for monitoring and diagnosis. US findings, including focal hypoechogenicity, tendon calcification, and neovascularization, have been reported to be correlated with the severity of tendinopathy^8–10^. As such, these US feature scores have been not only applied as a reliable tool for quantifying such abnormalities in tendinopathy^11^, but are also regarded as essential parameters in animal models^12^. The tendon structure is primarily composed of an extracellular matrix (ECM), and thus it is thought that the biomechanical properties and US feature scores reflect ECM composition. However, there have been no studies addressing the correlation between these two evaluation modalities.

US has recently gained popularity as a non-invasive method for inferring the biomechanical properties of tendons *in vivo*. For example, elastography can be used to measure the strain distributions during a given compression force, allowing the stiffness of a tendon to be indirectly estimated^13^. However, elastography has a limitation in that it cannot measure soft tissues under large deformation^14–16^. This is important in the context of the current study, as tendinopathy may result from large deformations in tendons that occur during daily activities. The amplitude of echo intensity, due to the acoustoelastic effect, is related to alternations of stress and strain in biomechanical testing^17^. With increases of tension^18^ and strain level^19^ in the tendon, the amplitude of echo intensity increases. Moreover, the amplitude of echo intensity increases with increasing stress in cyclic testing^19^. It is imperative to establish the relationship between biomechanical properties and acoustoelastic information obtained via US to provide another measurement method of biomechanical properties. An US model for tendinopathy would offer a valuable tool for the *in vivo* monitoring of tendinopathy and the monitoring of the response to treatment without the requirement for biopsy with the inherent risks and regulatory hurdles. Therefore, we hypothesized that, in a collagenase-induced tendinopathy model of the rat Achilles tendon, US feature scores and echo intensity would be associated with biomechanical properties. We investigated the dynamic changes of histopathology, biomechanical properties, echo intensity, and US feature scores in the collagenase-induced tendinopathy model, and examined the associations among the biomechanical properties, echo intensity, and US feature scores.

## Results

### Histopathological results

**Figure 1:**
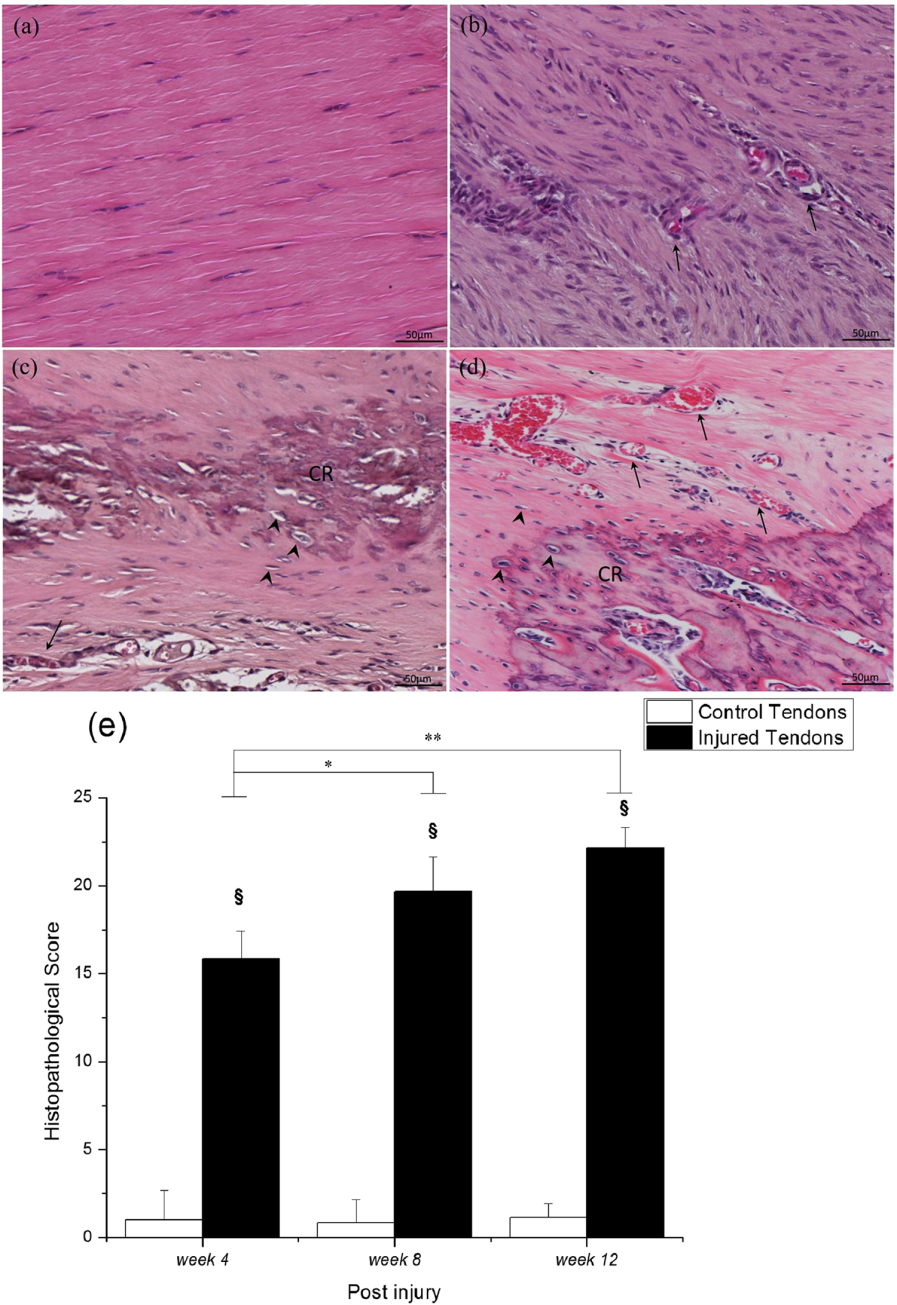
Hematoxylin-eosin staining and histopathological score of Achilles tendons. (**a**) Control tendon, and injured tendons at (**b**) *week 4*, (**c**) *week 8*, and (**d**) *week 12*. The summary of histopathological score along the healing time (**e**). CR, calcified deposits; arrowhead, chondrocyte-like cells; arrow, vascular structure. Bar in H&E = 50 *μ*m. ^§^compared with the control tendons; ^*/**^compared within the injured tendons; ^§/*^p < 0.05, ^**^p < 0.01.

Under a light microscope, we used a semi-quantitative scoring system modified from a previous study^20^ with a maximum total score of 24 points. The histopathological scores for the injured tendons were significantly higher than those of the control tendons throughout the study (all p < 0.05). For the injured tendons, the histopathological scores significantly increased with time: 15.83 ± 1.60 at *week 4*, 19.67 ± 1.97 at *week 8*, and 22.17 ± 1.17 at *week 12* (significant difference between *week 4* and *week 8*, p = 0.027; and between *week 4* and *week 12*, p < 0.001; Fig. 1e). One of the histological hallmarks of calcified tendinopathy, calcific deposits, were first observed at *week 4* (Fig. 1b) and present in all samples at *week 8* and *week 12* (Fig. 1c,d). Chondrocyte-like cells, another characteristic of tendinopathy, usually surrounded the calcific deposits, and were first present at *week 4* consistent with a previous report^21^. Other characteristics of tendinopathy, including neovascularization, hypercellularity, and loss of matrix organization, were also present since *week 4*. The tendon failed to heal up to *week 12*. These changes were not present in the sham control group throughout the study. Tenocytes were well aligned within longitudinally arranged collagen fibrils in the control group.

### Biomechanical properties

**Figure 2:**
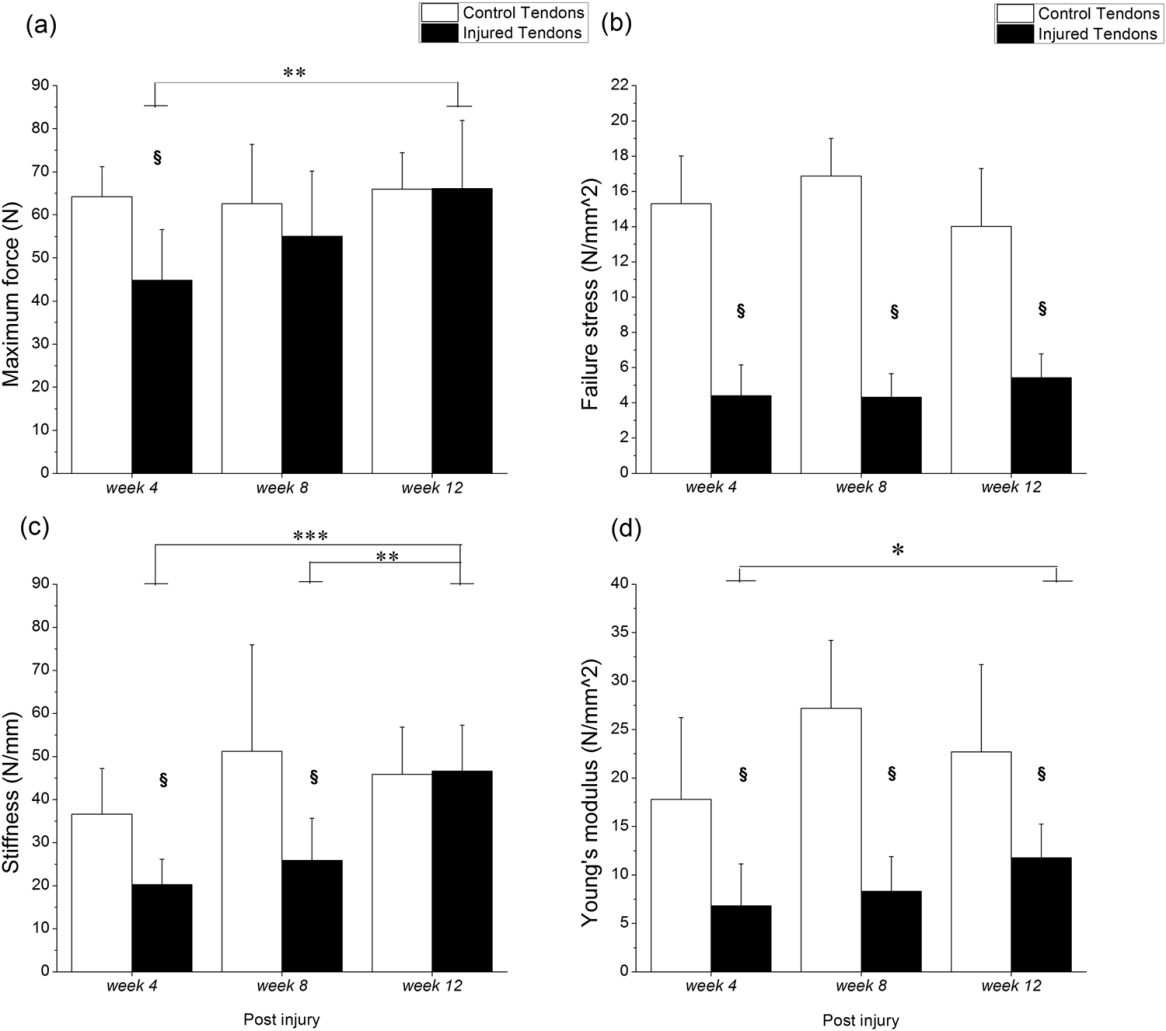
Biomechanical properties of Achilles tendons. (**a**) Maximum force, (**b**) failure stress, (**c**) stiffness, (**d**) Young’s modulus are shown after *week 4*, *week 8*, and *week 12*. ^§^Compared with the control tendons; ^*/**/***^compared within the injured tendons; ^§/*^p < 0.05, ^**^p < 0.01, ^***^p < 0.001.

The Achilles tendon was used to perform the biomechanical failure testing at a rate of 0.1 mm·s^−1^. The axial force-displacement and the stress-strain curves were plotted to obtain parameters of biomechanical properties. Compared with the cross-sectional area (CSA) of control tendons, that of injured tendons was consistently higher throughout the study (p < 0.05, Table 1). Therefore, compared with the failure stress of control tendons, that of injured tendons was consistently significantly lower at each time point (all p < 0.05, Fig. 2b) even though the maximum force in the injured tendons was lower only at *week 4* (p = 0.012, Fig. 2a). The Young’s modulus of the injured tendons was significantly lower at each time point (all p < 0.05; Fig. 2d) compared to that of the controls. The stiffness of the injured tendons was significantly lower at *week 4* (p = 0.025) and *week 8* (p = 0.012, Fig. 2c) compared to that of the control ones. During the healing progression after the index procedure, the maximum force and Young’s modulus in the injured tendons both increased, with significant differences between *week 4* and *week 12* (p = 0.008, and p = 0.015, respectively; Fig. 2a,d). Similarly, a significant increase in stiffness was found between *week 4* and *week 12* (p < 0.001) and between *week 8* and *week 12* (p = 0.005, Fig. 2c) for the injured tendons. There was no significant difference in the failure strain between control tendons and injured tendons, and also within time points post-injury in the control tendons. The failure strain was significantly lower at *week* 8 compared with *week 4* for the injured tendons (p < 0.05, Table 1). The range of failure strain is similar to the finding of previous study during the biomechanical failure testing at a rate of 0.1 mm·s^−1^ in a rat Achilles tendon^22^.

**Table 1:** Failure strain and cross-sectional area values.

	Group	*week 4*	*week 8*	*week 12*
Failure strain (%)	Control tendons	99.00 (18.77)	83.57 (30.94)	89.07 (19.98)
	Injured tendons	100.59 (23.80)	77.81 (18.85)^*^	85.27 (30.74)
Cross-sectional area (mm^2^)	Control tendons	4.27 (0.61)	4.01 (0.22)	4.85 (0.95)
	Injured tendons	11.46 (4.69)^§^	13.43 (3.69)^§^	12.67 (3.72)^§^

Values are expressed as means ± standard deviation.

^§^compared with the control tendons; ^*^significant difference between *week 4* and *week 8* in the injured tendons; ^§/*^p < 0.05.

### Ultrasound feature scores

**Figure 3:**
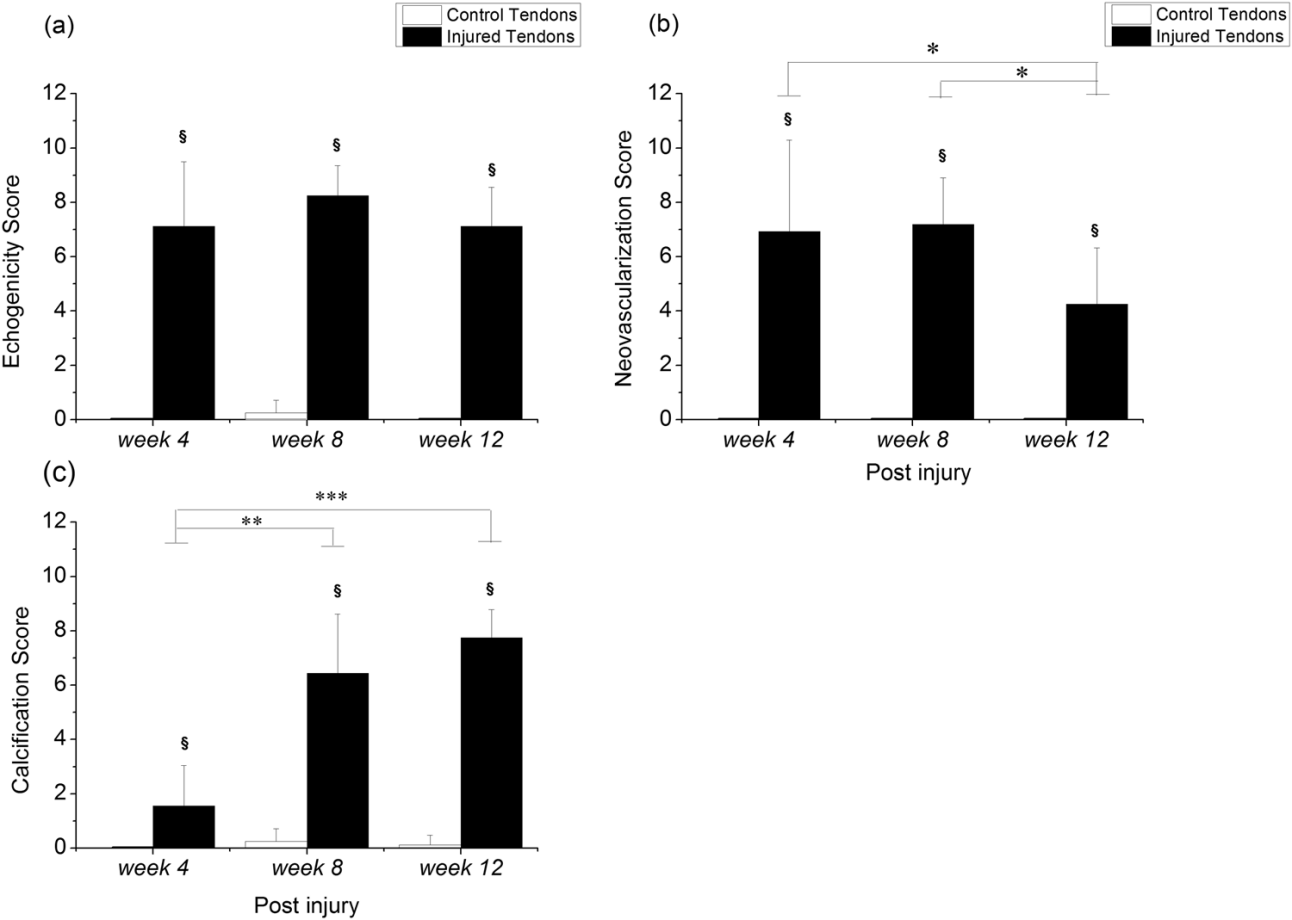
Ultrasound feature scores in Achilles tendons. ^§^Compared with the control tendons; ^*/**/***^compared within the injured tendons; ^§/*^p < 0.05, ^**^p < 0.01, ^***^p < 0.001.

The US features of the Achilles tendon were evaluated using a high-frequency US system *in vivo* prior to sacrifice. The levels of echogenicity, neovascularization, and calcification were scored from 0 to 10 based on a previous study^23^. The echogenicity, neovascularization, and calcification scores were significantly higher for the injured tendons than for the controls at *weeks 4*, *8*, and *12* (all p < 0.05, Fig. 3). For the injured tendons, there was no significant difference in the echogenicity scores among all time points following the index procedure (Fig. 3a). The neovascularization scores were significantly lower at *week 12* than at *week 4* (p = 0.026) or *week 8* (p = 0.022, Fig. 3b). The calcification score increased with healing progression post-injury, with a significantly higher score at *week 8* (p = 0.003) and *week 12* (p < 0.001, Fig. 3c) than at *week 4*. For the control tendons, there were no such changes in echogenicity, neovascularization, and calcification with healing time throughout the study.

### Echo intensity

**Figure 4:**
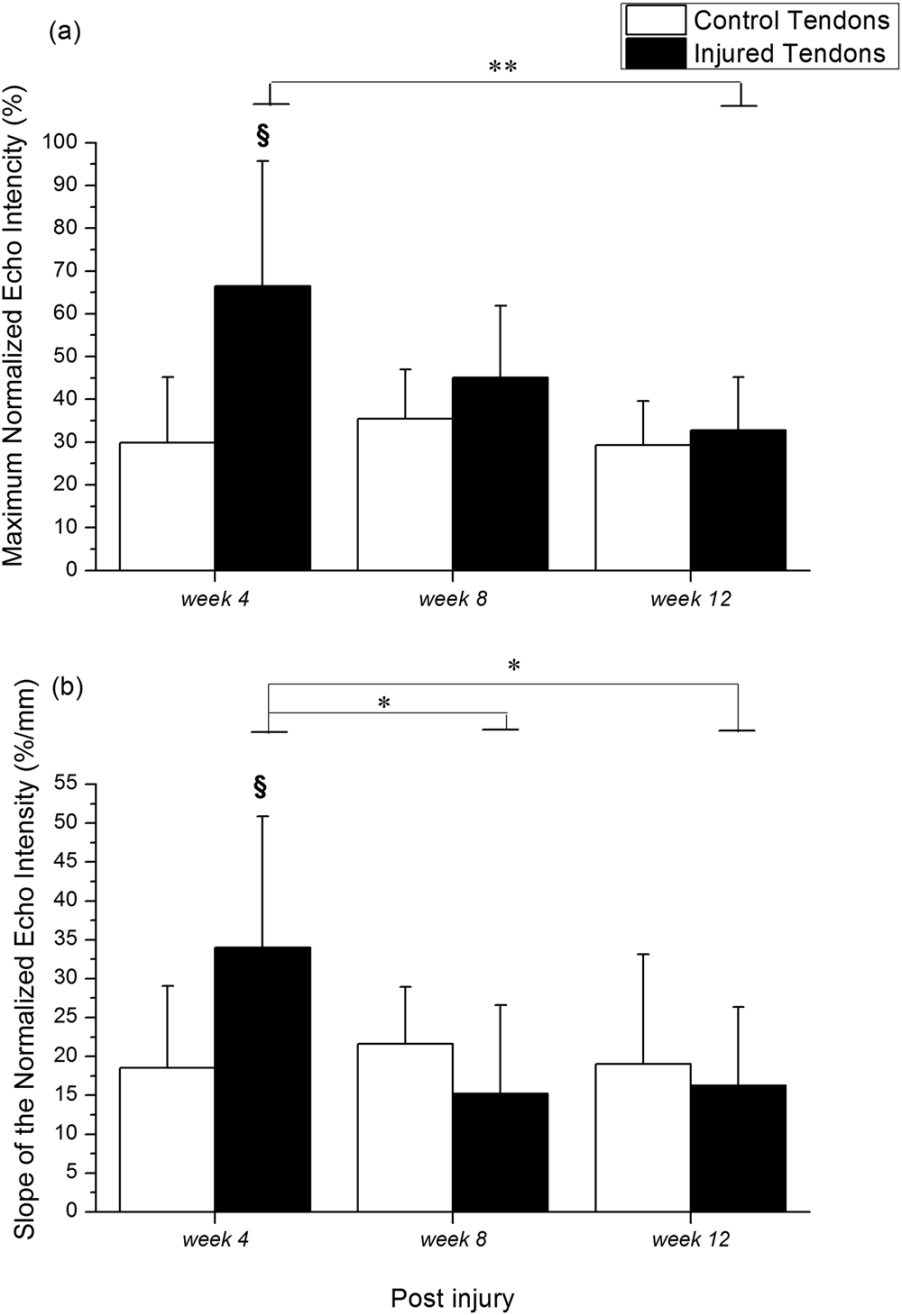
Normalized echo intensity of Achilles tendon. (**a**) Maximum normalized echo intensity, and (**b**) slope of the normalized echo intensity are shown after *week 4*, *week 8*, and *week 12*. ^§^Compared with the control tendons; ^*/**^compared within the injured tendons; ^§/*^p < 0.05, ^**^p < 0.01.

The echo intensity, the grayscale intensity of the region of interest (ROI), was recorded via US images during biomechanical failure testing. The maximum normalized echo intensity and the slope of the normalized echo intensity were analyzed based on the normalized echo intensity-displacement curve. The maximum normalized echo intensity value of the injured tendons was significantly higher than that of the control group at *week 4* (p = 0.012, Fig. 4a). The slope of the normalized echo intensity also showed a significant increase for the injured tendons compared to the control ones at *week 4* (p = 0.025, Fig. 4b). During healing progression after the index procedure, the maximum normalized echo intensity value at *week 12* was significantly lower than that at *week 4* (p = 0.005, Fig. 4a) for the injured tendons. The slope of the normalized echo intensity had a significantly greater value at *week 4* compared to *week 8* (p = 0.018) and *week 12* (p = 0.032, Fig. 4b) for the injured tendons.

### Correlation between biomechanical properties and ultrasonography

Pearson correlation was used to determine the correlation between the biomechanical properties and US characteristics in the injured and control groups. For the injured tendons, the stiffness had significant correlations with the maximum normalized echo intensity, neovascularization score, and calcification score (all p < 0.05, Table 2). Moreover, the failure stress had significant correlations with the echogenicity and neovascularization scores (both p < 0.001, Table 2). Significant correlations were also found between Young’s modulus and the echogenicity score, and between Young’s modulus and the neovascularization score (both p < 0.05, Table 2). The maximum strain had significant correlations with the maximum normalized echo intensity, slope of the normalized echo intensity, echogenicity score and calcification score (all p < 0.05, Table 2). Moreover, the maximum normalized echo intensity had significant correlations with the slope of the normalized echo intensity and calcification score (all p < 0.001, Table 2). The slope of the normalized echo intensity had significant correlations with the echogenicity and calcification scores (both p < 0.05, Table 2). No US or echo intensity features were correlated with the biomechanical parameters for the control tendons.

**Table 2:** Correlation coefficients between biomechanical parameters and characteristics of ultrasonography in the injured tendons.

	Maximum normalized echo intensity		Slope of the normalized echo intensity		US feature scores					
	*r*	p value	*r*	p value	Echogenicity		Neovascularization		Calcification	
					*r*	p value	*r*	p value	*r*	p value
**Biomechanical parameters**										
Maximum force	−0.238	0.262	−0.246	0.247	0.137	0.523	−0.324	0.131	0.343	0.101
Stiffness	−0.444^*^	0.030	−0.116	0.589	−0.306	0.145	−0.585^**^	0.003	0.417^*^	0.043
Failure stress	0.020	0.926	0.322	0.125	−0.685^***^	<0.001	−0.705^***^	<0.001	−0.178	0.407
Young’s modulus	−0.332	0.113	−0.112	0.602	−0.493^*^	0.014	−0.670^***^	<0.001	0.195	0.362
Maximum Strain	0.513^*^	0.010	0.582^**^	0.003	−0.470^*^	0.021	−0.032	0.884	−0.446^*^	0.029
**Echo intensity**										
Maximum normalized echo intensity	—	—	0.705^***^	<0.001	−0.125	0.560	0.220	0.314	−0.656^***^	<0.001
Slope of the normalized echo intensity	0.705^***^	<0.001	—	—	−0.465^*^	0.022	−0.074	0.737	−0.728^***^	<0.001

*r* is correlation coefficient; ^*^p < 0.05, ^**^p < 0.01, ^***^p < 0.001.

### Association between biomechanical properties and ultrasonography

The linear regression model was used to analyze the association between biomechanical parameters and the selected US characteristics in the injured and control groups. For the injured tendons, the maximum normalized echo intensity had a significant positive linear association with the maximum strain (p = 0.024) after adjustments for other US or echo intensity features (Table 3). The neovascularization score had a significant negative linear association with failure stress and Young’s modulus (Table 3). The US echogenicity score had no association with the biomechanical parameters. No US or echo intensity features were associated with the biomechanical parameters for the control tendons.

**Table 3:** Association between biomechanical parameters and characteristics of ultrasonography in the injured tendons.

Biomechanical parameters	Maximum normalized echo intensity	US feature scores	
		Echogenicity	Neovascularization
Maximum force			
*β*-coefficient	−0.145	2.692	−2.617
95%CI	(−0.584, 0.294)	(−2.638, 8.022)	(−5.951, 0.716)
p value	0.498	0.304	0.117
Stiffness			
*β*-coefficient	−0.312	−1.899	−2.066
95%CI	(−0.631, 0.006)	(−5.769, 1.970)	(−4.486, 0.354)
p value	0.054	0.317	0.090
Failure stress			
*β*-coefficient	0.015	−0.295	−0.331
95%CI	(−0.012, 0.041)	(−0.617, 0.027)	(−0.533, −0.130)
p value	0.259	0.070	0.003^**^
Young’s modulus			
*β*-coefficient	−0.070	−0.915	−0.663
95%CI	(−0.156, 0.016)	(−1.955, 0.126)	(−1.314, −0.012)
p value	0.104	0.082	0.046^*^
Maximum strain			
*β*-coefficient	0.007	−0.053	0.003
95%CI	(0.001, 0.012)	(−0.122, 0.017)	(−0.041, 0.046)
p value	0.024^*^	0.131	0.894

The *β*-coefficient and the corresponding 95% CI were estimated from the linear regression model; ^*^p < 0.05, ^**^p < 0.01.

## Discussion

This is the first study to evaluate the dynamic changes in the histopathology, biomechanical properties, echo intensity, and US feature scores in a collagenase-induced tendinopathy model. We also examined the associations among the biomechanical properties, echo intensity, and US feature scores. Our findings indicate that for the model of US-guided collagenase injection into rat Achilles tendons, higher histopathological scores, lower Young’s modulus, and higher US feature scores were obtained than those for the control tendons throughout the study. Up to *week 12*, the injured tendons still showed the histopathological hallmark of tendinopathy and lower Young’s modulus/failure stress, indicating failure of tendon healing. In the linear regression model, the neovascularization scores had a significant negative linear association with the failure stress and Young’s modulus, while the maximum normalized echo intensity had a significant positive linear association with maximum strain, after adjustments for other US features and echo intensity.

Histologically, tendinopathy models should represent a defective healing response with the following characteristics: hypercellularity, neovascularity, ECM degradation, rounding of cell nuclei, and acquisition of chondrocyte phenotypes^12^. These features were all observed in our collagenase-induced model at *week 8* and *week 12*. Our results were similar to those in previous studies^21, 24^. Poor ECM organization may lead to decreased biomechanical properties of the injured tendons^25, 26^, and therefore susceptibility to further injury. Shah *et al*. reported a reductions of 48% in ultimate tensile stress and 59% in modulus for the injured legs compared to contralateral control legs at four weeks post-injury after collagenase injection^26^. Our results also showed significantly lower failure stress, stiffness, and Young’s modulus for the injured tendons compared to those for the control tendons throughout the study, which is consistent with previous studies^26, 27^. The CSA of injured tendons was consistently higher than the controls throughout the study, which may be due to the increased activity of the tenocytes and proliferation of the tissue^26^. Furthermore, previous research showed that the values of maximum load to failure and stiffness increased from day 7 to 28 following collagenase injection^26, 28^. In our study, the maximum force, stiffness, and Young’s modulus also increased with time in the collagenase-injected tendons, and were significantly higher at *week 12* than at *week 4*. Our results indicate that the mechanical properties of the injured tendons improved with time post-injury, and that this was associated with the healing, regeneration, and remodeling process of tendon injury during this period. However, up to *week 12*, the collagenase-injected tendons still showed defective healing, corresponding to the significantly lower failure stress and Young’s modulus compared to those for the control tendons. According to the results of biomechanical properties and histological characteristics, the collagenase-induced rat model used in this study presented defective healing, a hallmark of tendinopathy, and the corresponding changes in US features and biomechanical properties.

US can provide structural information, such as tendon thickness, tendon alignment, calcification, tendon echogenicity and tendon tears, for patients with tendinopathy^10, 11^. In this study, the calcification scores for the injured tendons increased with time after the index procedure, consistent with the histopathological changes reported by Liu *et al*.^21^. However, the presence of ectopic calcification was steady since *week 8* in our study, earlier than that observed by Liu *et al*. (*week 12*)^21^. There are two possible reasons for this. One is the different target tendon choice. The other is that the US-guided injection may increase the precision of injection, thus improving the consistency of the experimental protocol. The US features were consistent with the histological findings in the current study, which showed that neoangiogenesis improved and that severe calcification remained until *week 12*. Furthermore, the echogenicity scores were not significantly different throughout our study. This is one common criticism of the collagenase-induced model, which induces acute and severe inflammation in the early stages, after which the injured tendons enter the repair process. The disorganized fibers and edema associated with this approach may reduce echogenicity due to changes in the density of collagen and the interfaces of fibers. Therefore, the echogenicity scores were consistently high from *week 4* to *week 12*, and not associated with biomechanical parameters after adjustment for other US and echo intensity features. However, the echogenicity score was correlated with failure stress, Young’s modulus, and maximum strain. A previous study showed that echogenicity measured under a given tendon tension is positively correlated with maximum stress and elastic modulus in a naturally diseased lesion tendon^29^. In addition, the ECM of the tendon had a positive linear regression with stiffness in the healing process after surgical transection, as determined by measuring the tendon brightness^30^. In our study, the semi-quantitative echogenicity, the level of low echo intensity, had a negative correlation with failure stress and Young’s modulus. This indicates that the high echo intensity would accompany the increase of failure stress and Young’s modulus, which is similar to findings reported in previous studies^29, 30^. Consequently, the scoring of US features, including echogenicity, calcification, and neovascularization, provide a valuable assessments in the tendinopathy model^12^, and thus have high reliability clinically^11^.

The amplitude of echo intensity is a strain-dependent parameter that is linearly proportional to the strain level during cyclic loading^18^, and is also a time-dependent parameter^19^. A previous study showed that an increased overstretch strain level could induce a decrease in the echo intensity changes, and so a higher strain change is required during the mechanical testing to reach the same level of echo intensity changes following severe diffuse damage^31^. In this study, the maximum normalized echo intensity and slope of the normalized echo intensity were found to be positively correlated to the maximum strain in the injured tendon. This shows that the injured tendon requires a higher strain change to reach the same level of maximum normalized echo intensity and slope of the normalized echo intensity with tissue healing time. Nevertheless, the maximum normalized echo intensity and stiffness showed a negative correlation over time post-injury in the injured tendon. This suggests that the increased stiffness causes less strain change, which may induce a lower maximum normalized echo intensity. Young’s modulus is calculated from the linear slope of the stress-strain curve; however, there was no correlation between the maximum normalized echo intensity and Young’s modulus in this study, which may be due to the unobvious increase in the Young’s modulus with healing time (*week 8* vs. *week 12*) in the injured tendons. In addition, a previous study showed that the echo intensity is positively correlated with the slope of the stress-strain curve and stress in the healing tendon after surgical transection at a constant failure strain^32^. Because our study used the collagenase-induced tendinopathy model and a non-constant failure strain in the biomechanical testing, the above-mentioned factors may explain the differences in findings compared with a previous study^32^. Nevertheless, calcification may also affect the maximum normalized echo intensity, slope of the normalized echo intensity, and stiffness in collagenase-induced tendinopathy. Therefore, a greater degree of calcification resulted in greater stiffness and lower maximum normalized echo intensity and slope of the normalized echo intensity. Based on the correlation findings, the slope of the normalized echo intensity could be a substitute for the echogenicity score and the calcification score, and thus help US operators reduce scoring US features bias in clinics. Establishing a correlation among the echo intensity, biomechanical parameters, and scoring US features in the calcified tendinopathy model could assist clinicians in evaluating the biomechanical properties and US feature scores in tendinopathic tendons by using a quantified US-based method during a tendon lengthening process *in vivo*.

According to the linear regression model, and after adjustment for other US and echo intensity features when appropriate, the neovascularization score showed a significant negative association with failure stress and Young’s modulus. The maximum normalized echo intensity had a significant positive association with maximum strain. Our results suggest that neovascularization and the maximum normalized echo intensity are critical factors associated with mechanically altered tendinopathic tendons. The comprehension of the changes in the biomechanical properties and US characteristics during the healing process, as found in this study, could help clinicians understand the progression of pathology and existence of correlations between the biomechanical properties and US characteristics in tendinopathy, and also help them decide more appropriate treatments based on non-invasive US measurements.

There are some limitations to this study. First, the measurement of echo intensity, carried out using *in vitro* protocols in this work, is different from clinical *in vivo* practices. The associations and correlations among mechanical properties, echo intensity, and US feature scores thus need to be validated *in vivo* and the velocity of tendon displacement needs to be controlled during a tendon lengthening process *in vivo*. Second, the US calcification score was not included in the linear regression analysis due to its high correlation with echo intensity. Inclusion of the US calcification score would cause multicollinearity in the statistical analysis. Calcification is not always present in tendinopathy even though it is a US feature in tendinopathy. Calcified tendinopathy has a prevalence of 2.7% to 22% in rotator cuff tendons^33^ and that of at least 5% in the Achilles tendon^34^ for athletes. Further studies are necessary to evaluate the association between US calcification score and other biomechanical parameters. Third, the sample size in each group was small. However, the number of experimental and control tendons was acceptable for an animal study, and adequately depicted the differences between groups.

In conclusion, the US-guided collagenase-induced tendinopathy model presented in this study could thus be suggested a regeneration and insufficient healing model because a lower Young’s modulus was present in the injured tendons at *week 12* compared to *week 4* and histopathological findings that indicate failing healing were present at *week 12*. In addition, neovascularization and the maximum normalized echo intensity were associated with a mechanically altered tendinopathic tendon based on the linear regression model. Therefore, the non-invasive US methodology presented in this work, including the echo intensity and US feature scores, may provide useful information about biomechanical properties in tendinopathy. However, further *in vivo* studies are needed to validate the correlations and associations among the US characteristics and biomechanical properties.

## Materials and Methods

### Ethics statement

All experimental rats were purchased from the Animal Center at National Cheng Kung University, and the following experiments were done in accordance with protocols approved by the Institutional Animal Care and Use Committee of National Cheng Kung University (protocol number: 102288).

### Collagenase-induced tendinopathy animal model

**Figure 5:**
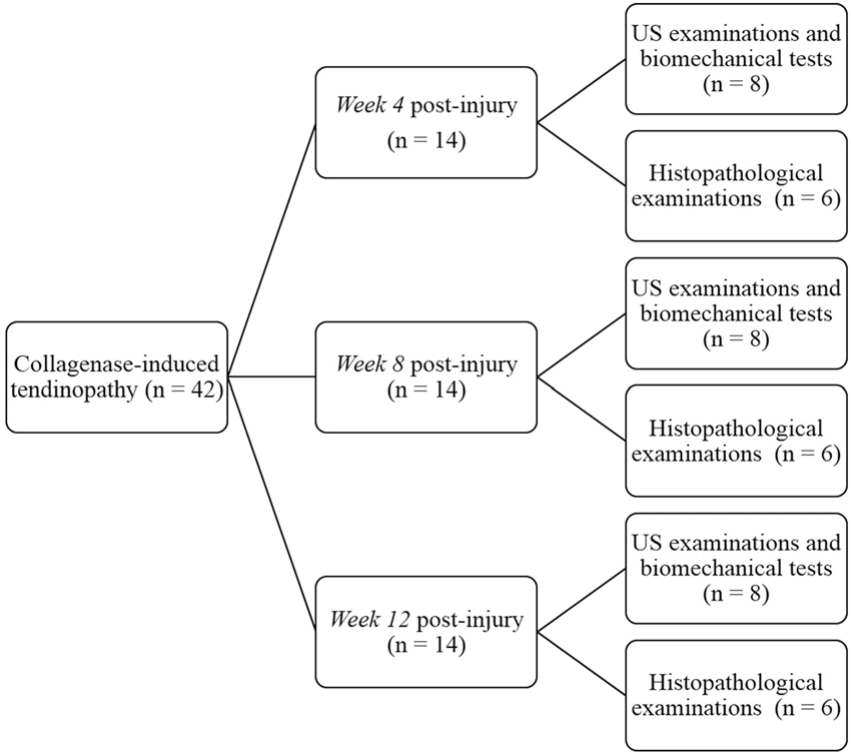
Schematic diagram of the experimental design.

Forty-two eight-week old male Sprague-Dawley rats weighing 290–330 g were used for the collagenase-induced tendinopathy model. After the collagenase-induced tendinopathy procedure, the forty-two rats were randomly assigned to one of three post-injury groups (n = 14 at each time point for post-injury group: *week 4*, *week 8*, and *week 12*; Fig. 5) by drawing lots, as done in a previous study^35^. For each rat, tendinopathy in the left Achilles tendon was induced by an intratendinous injection of 20 μL of bacterial collagenase I (Sigma-Aldrich, St Louis, Missouri) prepared by dissolving 0.015 mg/mL collagenase I in 0.9% saline^21^ followed by using a 29-gauge needle under US guidance (Vevo 770, VisualSonics, Toronto, Canada) with a 55-MHz linear transducer for high-resolution images to inject the collagenase into the same location within each tendon. The US guidance procedure was the same as that in our previous study^36^. The sham procedure involved needle puncture with a 29-gauge needle in the right Achilles tendon under US guidance, which was then set as the control tendon. After the above procedures, the rats were placed in a cage with free activity until *week 4*, *week 8*, and *week 12* post-injury, and then sacrificed using an overdose of pentobarbital before subsequent examinations. At each time point post-injury, 14 rats were randomly assigned to US examinations/biomechanical tests (n = 8) and histopathological examinations (n = 6) by drawing lots^35^.

### Scoring of US features and histopathological characteristics

The US features of the Achilles tendon were evaluated using a high-frequency (55 MHz) US system with *in vivo* at *week 4*, *week 8*, and *week 12* prior to sacrifice by an experienced orthopedic surgeon, who was blinded to the study protocol. The levels of echogenicity (hypoechoic area, 0: normal, 10: change throughout tendon), neovascularization(visible vessels, 0: none, 10: ≥10 vessels), and calcification (number of calcifications, 0: none, 10: ≥10 calcifications) were scored from 0 to 10 based on a previous study^23^. For histopathological examinations, both Achilles tendons in each rat were harvested from the muscle-tendon junction to the calcaneal insertion site. Each specimen was fixed in fresh 4% paraformaldehyde for 16–24 h at 4 °C, and then subsequently dehydrated, paraffin-embedded, and longitudinally sectioned. Sequential 4-μM sections were stained with hematoxylin and eosin. We used a semi-quantitative method to score each factor on a 4-point scoring system: 0 = normal, 1 = slightly abnormal, 2 = moderately abnormal, and 3 = markedly abnormal. The following parameters were modified from the previous study by Sullo *et al*.^20^ and assessed: fiber structure, fiber arrangement, roundness of the nuclei, regional variations in cellularity, increased neovascularization, decreased collagen stainability, fibrosis or hyalinization, and calcification characteristics. The maximum total score for each specimen was 24. Under a light microscope, one blinded examiner (IMJ) assessed the extent of tendinopathy.

### Biomechanical testing

The hamstring, Achilles tendon, and calcaneus were simultaneously removed after the 24 rats were sacrificed; the bone-Achilles tendon-muscle was then immersed in phosphate buffered saline (PBS) to keep the specimen hydrated. The specimen was used for the biomechanical testing and echo intensity measurement within 24 h. The tendon length, width, and thickness were measured three times using a digital image, and the CSA was calculated using the equation for the area of an ellipse. Following the CSA measurement, the specimen was carefully mounted on the material testing system (Tytron^TM^ 250, MTS Systems Corporation, Eden Prairie, USA) to prevent twists in the tendon using a custom-designed clamp. The specimen was immersed in a PBS bath and the temperature was controlled at 25 °C via a temperature regulator throughout the overall biomechanical testing in order to keep the specimen moist and at a constant temperature, and to provide the medium needed for the US examination. The specimen was preconditioned to a 0.1% strain (≈1 N)^26^ at 0.5 Hz for 20 cycles prior to being subjected to a static tensile pre-load of 0.1 N. After giving a static tensile pre-load of 0.1 N, biomechanical failure testing was performed on the tendons at a rate of 0.1 mm·s^−1^. The sampling rate was set at 100 Hz for biomechanical failure testing.

The initial length was defined as the grip length at a static tensile pre-load of 0.1 N. The axial displacement of the material testing system, which indicated the displacement between the grips, was recorded and normalized by the initial length of the tendon to calculate the percentage of tendon elongation, and this was presented as the strain. The axial force was divided by the CSA in the tendon, and this was presented as the stress. The maximum force was recorded during the process of failure testing and the stiffness was calculated from the linear slope of the axial force-axial displacement curve. The maximum strain corresponded to the maximum force. Based on the data of stress and strain, the stress-strain curve was plotted and the linear portion was fitted to calculate the Young’s modulus. The failure stress was defined as the maximum stress during the process of failure testing in the stress-strain curve.

### Echo intensity measurement

**Figure 6:**
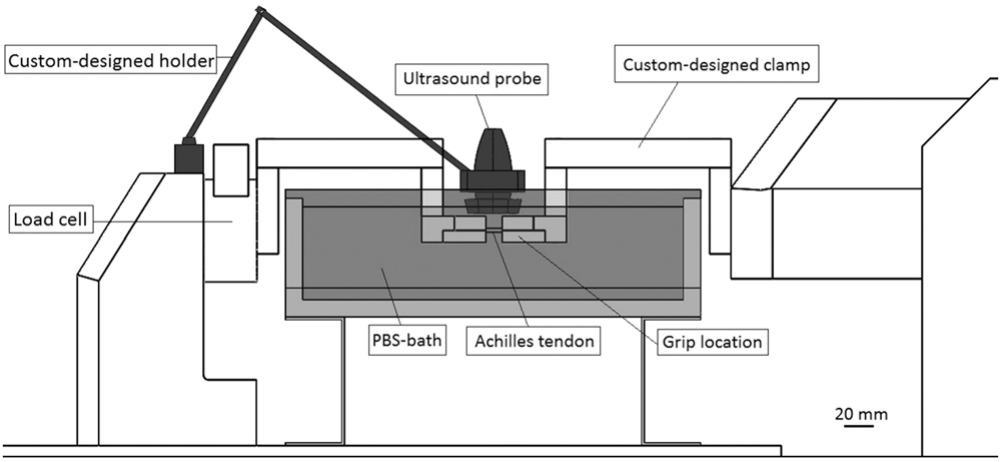
Experimental setup to measure biomechanics and echo intensity simultaneously. Bar = 20 mm.

The echo intensity in the B-mode ultrasonographic images was recorded simultaneously with a sampling rate of 25 frames·s^−1^ during the biomechanical failure testing (Fig. 6) via a 12L5 Linear Array Transducer at 12 MHz (Terason t3000^TM^, Teratech Corporation, Burlington, USA). The probe was held over the PBS bath by a custom-designed holder and oriented parallel to the tendon with a distance of 1.2 cm. The US setting was consistent through all the testing, so as to ensure standardized images for all specimens.

The normalized echo intensity was defined as the gray scale intensity in every frame related to the first frame in the ROI between the grips based on the US images via ImageJ (NIH) software. The normalized echo intensity-axial displacement curve was plotted to determine the maximum value of normalized echo intensity when the maximum force occurred, and the slope of the normalized echo intensity-displacement curve corresponded to the slope of axial force-axial displacement curve.

### Statistical analysis

A Wilcoxon signed-rank test was used to assess the differences in the histopathological, biomechanical and ultrasonographic results between the injured and control tendons at *weeks 4*, *8*, and *12* respectively. Differences in the histopathological characteristics, biomechanical parameters, echo intensity, and US feature scores with healing time within the same group were assessed using a Kruskal-Wallis H test, and post hoc analysis was performed with the Dunn method. For the tests of correlation and association among the different parameters, all data within the same group (n = 24) were pooled together for further analyses. The Shapiro-Wilk test was done for the normality of data and showed normal distribution in all parameters (Supplementary Table 1). Pearson correlation was used to determine the correlation among the biomechanical properties, echo intensity, and US feature scores. The association of biomechanical parameters with the selected US and echo intensity features (echogenicity score, neovascularization score and maximum normalized echo intensity) was analyzed using the linear regression model. The calcification score and slope of the normalized echo intensity were not included in this linear regression model, due to their high correlation with the other US or echo intensity features (*r* > 0.6 or <−0.6). Inclusion of the two parameters would cause the statistical problem of multicollinearity. The level of significance was set at p < 0.05. SPSS 17.0 statistical software (SPSS Inc., Chicago, IL, USA) was used for statistical analysis.

## Electronic supplementary material

Supplementary Table 1.
