# Supplementary material for: Characteristics of Sonography in a Rat Achilles Tendinopathy Model: Possible Non-invasive Predictors of Biomechanics
Source: Sci Rep. 2017 Jul 11;7:5100. doi: 10.1038/s41598-017-05466-y (PMC5506063; doi:10.1038/s41598-017-05466-y)
Supplement: Supplementary file 1 — Supplementary Table 1. [file 41598_2017_5466_MOESM1_ESM.doc]

**Characteristics of Sonography in a Rat Achilles Tendinopathy Model: Possible Non-invasive Predictor of Biomechanics**

Su-Ya Lee^1^, Hsiao-Feng Chieh^1,2^, Chien-Ju Lin^2,1^, I-Ming Jou^3,4^, Yung-Nien Sun^5,2^, Li-Chieh Kuo^6,2^, Po-Ting Wu^4,7,1,2^*, Fong-Chin Su^1,2^*

^1^Department of Biomedical Engineering, National Cheng Kung University, Tainan, Taiwan

^2^Medical Device Innovation Center, National Cheng Kung University, Tainan, Taiwan

^3^Department of Orthopedics, E-Da Hospital, Kaohsiung, Taiwan

^4^Department of Orthopedics, College of Medicine, National Cheng Kung University, Tainan, Taiwan

^5^Department of Computer Science & Information Engineering, National Cheng Kung University, Tainan, Taiwan

^6^Department of Occupational Therapy, National Cheng Kung University, Tainan, Taiwan

^7^ Department of Orthopedics, National Cheng Kung University Hospital, College of Medicine, National Cheng Kung University, Tainan, Taiwan

***Corresponding authors (Po-Ting Wu and Fong-Chin Su equally contributed to this work)**

Po-Ting Wu, M.D., Ph.D.

Department of Orthopedics, College of Medicine,

National Cheng Kung University,

No. 1 University Road,

Tainan 701, Taiwan

Tel: +886-6-2353535 ext. 5237

Fax: +886-6-2766189

Email: anotherme500@gmail.com

Fong-Chin Su, Ph.D.

Department of Biomedical Engineering,

National Cheng Kung University,

No. 1 University Road

Tainan 701, Taiwan

Tel: +886-6-2757575 ext. 63422

Fax: +886-6-2376604

Email: fcsu@mail.ncku.edu.tw

Supplementary Table 1. The normal distribution testing in the injured tendons.

| Biomechanical parameters | p value |
| --- | --- |
| Maximum force | 0.636 |
| Stiffness | 0.454 |
| Failure stress | 0.897 |
| Young’s modulus | 0.280 |
| Echo intensity |  |
| Maximum normalized echo intensity | 0.347 |
| Slope of normalized echo intensity | 0.073 |
| US feature scores |  |
| Echogenicity | 0.240 |
| Neovascularization | 0.384 |
| Calcification | 0.120 |
